# Psychosocial factors affecting the bereavement experience of relatives of palliative-stage cancer patients: a systematic review

**DOI:** 10.1186/s12904-022-01096-y

**Published:** 2022-11-30

**Authors:** Marie Hasdenteufel, Bruno Quintard

**Affiliations:** Laboratoire de Psychologie EA 4139, Université de Bordeaux, Faculté de Psychologie, 3 Ter, Place de La Victoire, 33076 Bordeaux-Cedex, France

**Keywords:** Caregivers, Relatives, oncology, Palliative care, end-of-life, Mourning, Grief, Bereavement

## Abstract

**Background:**

Cancer is one of the leading causes of death worldwide and a cancer death is a major risk factor for pathological bereavement. This systematic review of the literature aimed to identify biopsychosocial and existential determinants specific to the palliative phase of cancer that influence the grieving experience of the caregiving relative.

**Method:**

A systematic review of the literature was conducted without language or time restrictions. The Cairn, Cochrane Library, PubMed, PsycArticle, PsychInfo, Psychology and Behavioral Sciences Collection databases were explored. All studies assessing pre- and post-death measures and focusing on friends and relatives caring for adults with cancer in palliative care services were included in the review.

**Results:**

Out of 645 articles identified, 18 full text studies were finally included in our systematic review of the literature. Many factors specific to the cancer palliative phase were identified as influencing the bereavement experience of caregivers, with factors relating to: 1) the caregiver (e.g. social support, psychological burden, preparation for loss, action and discussion related to death); 2) the patient (e.g. denial or acceptance); 3) the interactions between patient and their caregivers (e.g. tensions, communication difficulties, and presence at the time of death); and 4) the end-of-life context. The caregiver's grief experience can be described by the following terms: typical and pathological grief, anxiety, depression, guilt, psychological distress, post-traumatic stress disorder and post-traumatic growth, and life satisfaction.

**Conclusions:**

Many contextual, sociodemographic, dispositional and transactional factors specific to the palliative cancer phase are involved in the caregiver's grieving experience. Avenues for reflection and recommendations are proposed including supporting communication and patient-relative relationships, evaluating the nature and degree of functionality of coping strategies, strengthening the robustness of methodologies, considering impact of COVID-19, and new lines of enquiry for research.

## Background

The end-of-life period represents a real upheaval, both for the patient and for relatives and friends [1–4] who are exposed to many disturbances: physical (e.g. asthenia, anorexia, etc.), social (e.g. isolation, precariousness, etc.), psychological (e.g. fears, impotence, exhaustion, depression, etc.) and family (e.g. communication difficulties, unspoken, conflictual relationships, etc.) [5]. These problems are all the more important as the physical degradation of the patient worsens and the fatal outcome gets closer [6]. At times, the distress of the family member can be greater than that of the patient [7, 8]. This new phase of distress may cause a real emotional uproar: sadness, guilt, shame, as well as anticipatory grief of who their loved one was, of a part of themselves, and of unrealized plans, etc. [6, 9].

After loss, the bereaved then enters the grieving process, a psychological process in which they learn to live with the loss of their loved one [10]. It’s a normal process. It leads the bereaved to the gradual acceptance of the reality of death, to the habituation of the absence of the loved one, to the acceptance of the changes that the loss has brought about in their life and in their world, and to the reorganization of their own internal models [10–12]. The grieving process includes cognitive, emotional, behavioral and physiological responses (Table 1) [10–13]. While grief is unique, in the image of the relationship between the bereaved and the deceased, certain time trends may emerge to define typical/normal grief [14]. These trends, however, must be considered with caution [14]. It has been proposed that about six months after death, acute feelings and negative emotional states give way to a gradual calming of emotions and there may be a sense of habituation in that all negative emotions and feelings have already been experienced at least once [11, 12]. After one year, a psychological journey will have been traveled [11, 12]. In general, distress tends to decline gradually and acceptance of death increases with time following loss [10, 15].Thus, grief can be thought of as an overlay of emotional states and behaviors whose associations and intensities vary according to the individual [11, 12]. The stages and temporal dynamics are only a general framework, supporting the need to understand ways to differentiate the typical from the pathological.

**Table 1:** Cognitive, emotional, behavioral and physiological reactions to grief

**Cognitive reactions**	**Emotional reactions**
Mental disorganization, intrusive images, idealization of the deceased, avoidance of distress, low self-esteem, etc. [10, 12, 16]	Shock, inoperability, sadness, lack, anxiety, anger, guilt, regret, feeling of emptiness, hopelessness, depression, feeling of loneliness, suspicion, relief, affliction, gloom, overwhelm, feeling of emptiness, feeling of absence, tensions, etc. [10–12, 16]
**Behavioral reactions**	**Physiological reactions**
Agitation, fatigue, crying, difficulty maintaining social interactions (maintenance, rejection, initiation), languishing of the deceased, imitation of the deceased's behavior, communication with the deceased increased use of psychotropic drugs, increased alcohol intake and tobacco, etc.[10, 12, 16]	Loss of appetite, bulimic behavior, insomnia, increased need for sleep, disturbed circadian rhythm, loss of energy, somatic complaints (headache, neck pain, muscle cramps, nausea, vomiting, heartburn, palpitations, tremors) hair loss, decreased immunity, stress-related pathologies, etc.[10, 16]

While no one emotion, behavior, or cognition is sufficient to assert that grief has become complicated, high intensity and abnormal duration of these manifestations can point the diagnosis towards pathological grief [12]. It is no longer just the pain and sorrow that lasts, but the quality of life and health that have deteriorated to the point of endangering the life of the bereaved [12]. It is then that grief becomes pathological. The terminology for qualifying pathological bereavement is vast. In this review only one term will be used, that of pathological grief. Previous criteria for diagnosing pathological grief were heterogeneous and without consensus [12]. The 2022 revision of the DSM-5marks a major turning point with Prolonged Grief Disorder now appearing alongside trauma and other stress-related disorders [17]. It is characterized by intense sadness, emotional pain and preoccupation with the death of the deceased, with other accompanying symptoms [17, 18]. Distress is significant and reactions are disproportionate with culture, religious or age norms [17, 18]. Twelve months of symptoms are required to establish such a diagnosis [17, 18]. The ICD-11 echoes the DSM-5 proposal with the mention: "prolonged grief disorder" [19]. It differs from DSM criteria in terms of duration; these symptoms must persist for an atypically long period of time after the loss, at least six months [19]. In summary, grief is described as "pathological" when corresponding to a lasting exacerbation of emotional distress and an important concern in relation to the deceased [18–21]. Pathological grief must be distinguished from a major depressive episode and a post-traumatic stress disorder. Compared to depression, pathological grief is characterized by a significant fluctuation in sadness, pleasant daydreams and the persistence of positive emotions related to the memory or evocation of the deceased. Compared to post-traumatic stress disorder, the sadness that is seen in pathological grief is more marked. In addition, the lack of nightmares and the fear of forgetting the deceased contrast with the presence of nightmares and the desire to forget the original trauma of post-traumatic stress disorder [12].Various risk factors for pathological grief have been identified in literature (Table 2) [12, 16, 22].

**Table 2:** Risk factors for pathological bereavement

Risk factors related to the deceased’s characteristics and to the circumstances of death	Loss of a child, a spouse, a sibling Death from cancer Traumatic context (e.g. suicide) Sudden death Low preparation for death (not just for suicide) Specificities induced by the disease and its treatments Deficient quality of care and end-of-life support Duration of relationship Conflicting and avoidant relationship Dysfunctional family dynamics Type of attachment to the deceased (insecure, anxious, dependent, ambivalent, disorganized) [10, 12, 16, 23–30]
Risk factors linked to the bereaved	Young and old Gender (Women) Low level of income Low level of education Childhood neglect and abuse Insecure attachment mode Separation anxiety History of depression, emotional disturbances, psychiatric pathologies Previous losses, difficult experiences, succession of difficult bereavements, unresolved old bereavements Low sense of internal control Coping of dysfunctional grief Low level of optimism Feeling of burden Impact of care on the caregiver's schedule Negative perception of the death situation Exhaustion Difficulties in carrying out daily activities Lack of religious beliefs Lack of social resources[10, 12, 16, 23–25, 27, 30–34]

These risk factors are common to the general population and have been identified without consideration of end-of-life trajectories nor type of death (sudden versus expected). However, several authors, including Murray (2005), have carried out work that highlights different end-of-life trajectories. Murray (2005) models three end-of-life trajectories according to the nature of the decline inherent to each pathology: cancer, organ failure, and neurodegenerative pathology [35]. Each of these trajectories is specific. We hypothesize that the experience of the bereaved differs according to a given trajectory. This is all the more plausible as we note that cancer appears among the risk factors for pathological bereavement, as well as the specificities induced by the disease and its treatments [10, 36–38]. In general, and to our knowledge, studies have not distinguished between these types of trajectories and when they do, they focus on neurodegenerative diseases. However, with 10 million deaths in 2020, cancer is one of the main causes of death in the world [39]. It therefore seems essential to understand the specificities of this end-of-life trajectory in order to identify the main risk factors for pathological bereavement. While the identification of risk factors is crucial, the identification of key protective factors is equally important. Taking these two factors into account would allow for more exhaustive modeling to support health professionals. Although the identification of protective factors is beginning to emerge in the literature, it remains limited [40]. Finally, the bereaved experience is currently only defined by a single categorical perspective: pathological (prolonged grief disorder) versus non-pathological (typical grief). For this reason we have attempted to integrate the different health outcomes related to the experience of the bereaved in order to move towards a slightly more dimensional approach.

In summary, little research to date has focused on the experience of bereavement following cancer from a diachronic perspective, exploring how the experience of the days preceding the loss might influence the experience of loss [41, 42]. The objective of this study is to carry out a systematic review of the literature to identify the main biopsychosocial and existential factors specific to the palliative phase of cancer (pre-loss) that can exert a favorable or unfavorable influence on caregivers’ experience of loss of a loved one (post-loss).

## Method

### Eligibility criteria, information sources and search strategies

This systematic review of the literature was conducted in the spirit of the Cochrane approach. In order to meet the research objective, we established eligibility criteria: PICO (Population, Intervention, Comparison, Outcomes) criteria; study plan; publication status language (Table 3) [43].

**Table 3:** Eligibility Criteria

**PICO**^a^ **Criteria**	
Population	A person 18 years of age or older, caring for a family member, friend or close friend in the palliative phase of cancer(cancer that is unlikely to be cured or controlled with treatment; the cancer may have spread from where it first started to nearby tissue, lymph nodes, or distant parts of the body; treatment may be given to help shrink the tumor, slow the of cancer cells, or relieve symptoms but it is not a cure [44]) being cared for by specialized palliative care services/units
Intervention	Information on grief adjustment related to the experience prior to the loss specific to the palliative phase
Comparison	Not applicable here
Outcome	Relative quantitative and qualitative measures: grieving process, quality of life, emotional functioning, intra- and inter-personnel functioning, cognitive functioning
Study plan	Randomized and non-randomized / observational Protocols: experimental, quasi-experimental, non-experimental (exclusion: case studies, conference summaries, expert opinions including interviews)
Methods	Quantitative Qualitative Mixed
Strategy	Interview surveys Questionnaire surveys Observational surveys (participant, non-participant, structured, unstructured observation)
Study design	Retrospective Prospective
Publication status& language	No restrictions

^a^*PICO* Population intervention comparison outcomes

Based on the eligibility criteria mentioned above, search strategies were conducted in the following databases: PubMed, PsycInfo, PsycArticles, Psychology and Behavorial Sciences, Cochrane Library, and Cairn.

While the search terms were the same for each database, they were adapted to the functionality of each. The search terms used with thesaurus functions were as follows: "Bereavement" AND "Family" AND “Terminally ill patient”. To account for nuances in some key terms (e.g. for the expression "Terminal Care"), the search terms used without thesaurus functions were: “family OR families OR relatives OR parents OR siblings” AND “terminal illness OR palliative OR end of life” AND “grief OR loss OR bereavement OR mourning ". Without the thesaurus, a search was carried out with "Keyword" and "Subject" descriptors. All of these terms were used in each database and were co-constructed and validated in consultation with a librarian. The search was last updated on 28 February 2022.

### Selection process and analyses

We followed the Cochrane recommendations in order to carry out the selection process. All search results were merged into an Excel spreadsheet. Duplicates were excluded. The title and abstract of each of the articles were reviewed. Those who did not meet the eligibility criteria were excluded. The process of excluding articles on the basis of title and abstract, was carried out independently by both authors on 10 references randomly selected in order to reinforce the validity of our investigation. Authors were asked to indicate whether the article was retained or was rejected. Agreement between authors had to be sufficient for one of the authors to perform the screening alone. Following initial exclusions, the full texts were studied. Those that then did not meet the eligibility criteria were excluded. In the same way, the process of excluding articles on the basis of reading the full texts, was carried out in independent double rating, by MH and BQ on 10 references randomly selected to reinforce the validity of our investigation. Authors were asked whether the article was included, rejected, or "required discussion. Agreement again had to be sufficient for one of the authors to perform the screening alone. For retained articles, a table of study characteristics was completed. The reference lists of all articles eligible for the systematic literature review were reviewed for inclusion in the review. The same approach was applied: review of titles and abstracts, review of the full text of the article; for those eligible for the review, important data from the article were included in the study characteristic table. This process was repeated until a redundancy and/or a total exclusion of all the references studied was achieved.

Articles eligible for review were qualitatively assessed using a study quality assessment grid [45]. This tool is easy to use and gives an overall score for the quality of a study from 0 to 32 points. The score was defined from a consensus obtained by 2 independent contributors (BQ and MH). The five parts included questions about: 1) study quality, 2) external validity, 3) study biases, 4) confusion and selection bias, and 5) study power.

A thematic synthesis was conducted separately by two psychology researchers (MH & BQ) who compared their respective results in order to identify the main themes in the literature.

### Study selection

**Fig. 1:**
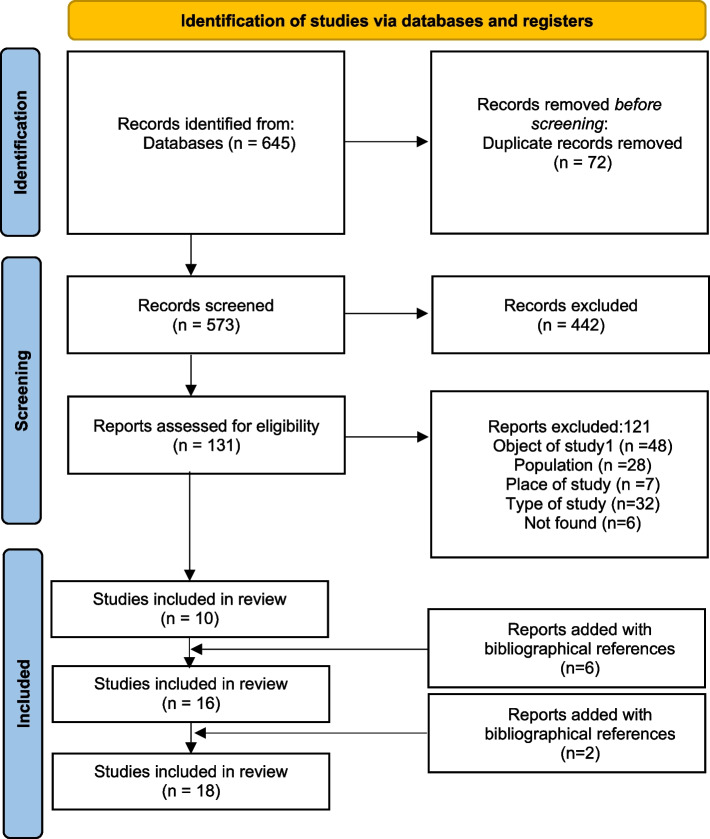
Flowchart of the study selection process

Six hundred and forty-five studies were found through our research strategy, including 214 from PsycInfo, 53 from Psychology and Behavioral Sciences Collection, 11 from PsycArticles, 233 from PubMed, 88 from Cochrane and 43 from Cairn (Fig. 1). After excluding duplicates (*n* = 72), 573 studies were studied on the basis of title and summary and 442 were excluded. As a result, 131 studies were analyzed in their entirety. One hundred and twenty-one studies were excluded and the 10 remaining studies included. References of the included studies were reviewed (*n* = 223) and 6 additional eligible studies were included. The references of these 6 studies were also reviewed (*n* = 119) and two further studies were identified for inclusion in our review. The study of the reference lists of these last 2 studies (*n* = 47) did not result in the inclusion of other studies. These were essentially duplicates and studies that were not relevant to our research objectives. Overall, 18 studies were included in our systematic review (Table 4).

**Table 4:** Characterist﻿ics of studies

Source	Design	Population	Mesures	Results	Qualitative Analysis
*Yamaguchi, Maeda, Hatano, Mori, Shima, Tsuneto, Kizawa, Morita, Yamaguchi, Aoyama&Miyashita, 2017 *[46] Effects of end-of-life discussions on the mental health of bereaved family members and quality of patient death and care	Transversal Retrospective Mean time between death and completion of the protocol = 283.4 days	Palliative care services, Japan *N* = 9123 Age: < 50: 18.8% 51–60: 25.7% 61–70:30.2% > 71:25.2% Woma*n* = 66.7% Spouse = 46.4% Primary cancer site - Lung 23.1% - Gastrointestinal tract: 45.8% - Breast: 4.9% - Others:29.3%	Sociodemographic data: -patient: physical condition, treatment preferences, need for information, awareness of terminal illness -caregiver: education, communication with the patient about the disease, treatment preferences, information needs End-of-life discussions Quality of Death (Good Death Inventory – Short Version) Quality of end-of-life care (Care Evaluation Scale—Short Version) Depression (Patient Health Questionnaire-9) Complicated grief (Brief Grief Questionnaire)	Discussions about the end of life are associated with lower prevalence of depression and complicated grief in bereaved The earlier discussions about the end of life took place, the lower the prevalence inherent in depression and complicated grief Discussions about the end of life are associated with a better perception (of bereaved) of the quality of death and end of life care	12/32
*Dumont, Dumont &Mongeau, 2008*[47] End-of-life care and the grievingprocess: Family caregivers who have experienced the loss of a terminal-phase cancer patient	Transversal Retrospective Average time between death and completion of the protocol: 137 days	Hospital specializing in palliative care, Quebec, Canada *N* = 18 Age [33—75 years old] Woma*N* = 61% Spouse = 66.6% Cancer	Semi-structured interview guide: building blocks of caregiving that could have facilitated or hindered the grieving process - Nature of attachment - Circumstances surrounding death - History (caregiver) - Personal data (caregiver) - Social data - Stress factors	Risk factors for adjustment to bereavement: - Caregiver: difficulty expressing feelings, presence of a psychological and emotional burden, unsatisfactory informal and formal support; - Patient: patient denial of his illness, aggressiveness, presence of symptoms (confusion, major changes in behavior, cachexia, uncontrollable pain), departure to a hospital or palliative care; - Caregiver-patient: communication problems, ambivalent or dependent relationship, presence of family tensions Protective elements for adjustment to bereavement: - Caregiver: optimism, ability to assert oneself, religious and spiritual beliefs, past experiences, satisfactory formal and informal support, preparation for loss; - Patient: acceptance of the disease, control of pain and suffering, respect for the sanctity of death and the dignity of the patient; - Caregiver-patient: presence of significant relationships, presence of the family at the time of death	9/32
*Kelly, Edwards, Synott, Neil, Baillie & Battistutta, 1999 *[43] Predictors of bereavement outcome for family carers of cancer patients	Longitudinal Prospective Time 1: before the patient's death Time 2: 122 days after the patient's death	Hospice Homecare Service at Mt Olivet Community Services, Brisbane, Australia *N* = 178 Age = 57 years old Woma*N* = 76% Spouse = 60% Cancer diagnosis: - Lung: 28% - GI: 32% - Breast: 7% -- Melanoma: 5% - Genito-urinary: 6% - Prostate: 5% - Gynaecologic: 3% - Haematologic: 2% - Brain:CNS: 2% - Other: 4% - Unknown primary: 6%	Caregiver Social support (Social Health and Social Contact Instrument) Number of adverse life experiences (Life Events Inventory) Ways of Coping Measure Perception by caregivers of the frequency of specific patient symptoms + distress they cause to caregivers Quality of the relationship between the caregiver and the patient (Intimate Bond Measure) Anxiety and Depression Symptoms (DSSI Anxiety and Depression Scale) Psychic distress (The General Health Questionnaire) Guilt Scale Post-traumatic stress disorder (Impact of Events Scale) Symptoms of bereavement (Bereavement Phenomenology Measure) Patient Functional state (Karnofsky Palliative Scale) Cognitive state (Mini Mental State) Quality of life (QL Index)	During step 1, as a caregiver, being a woman, having anxious depressive symptoms and a lower level of practical assistance, having a perception of a higher level of control in the caregiver-patient relationship, to identify a greater number of undesirable life events and that the patient be more seriously are so many elements which predict, at step 2, the anxiety and depressive symptoms in the bereaved In step 1, the psychic distress experienced by the caregiver predicts, in step 2, the bereavement symptoms in the bereaved In step 1, the caregiver's use of avoidance as a coping mechanism, a lower quality in the caregiver-patient relationship, and separation from parents in childhood predict, at step 2, post traumatic stress disorder During step 1, as a caregiver, the fact of experiencing a feeling of guilt, of having the perception of less attention to the patient-caregiver relationship, of experiencing a lower level of practical assistance, separation from the parents during childhood and a quality of life and a general state of health of the patient which appear to be altered, are so many elements which predict, at step 2, the feeling of guilt	10/32
*Hirooka, Fukahori, Taku, Togari& Ogawa, 2017 *[45] Quality of death, rumination, and posttraumatic growth among bereaved family members of cancer patients in home palliative care	Transversal Retrospective Mean time between death and protocol completio*N* = 912.5 days	Palliative home care services, Japan *N* = 805 Age = 63 years old Woma*N* = 71.8% Spouse = 52% Primary cancer sites: - Lung: 23.3% - Stomach: 16.7% - Pancreas: 9.5% - Colon: 9.4% - Others: 32%	Sociodemographic data: - patient: age, gender, 1st location of cancer - caregiver: age, gender, relationship with the patient, religious status Quality of Death (Good Death Inventory-Short Version) Post Traumatic Growth Inventory	Being woman as a caregiver predicts post-traumatic growth in the bereaved The caregiver's religious beliefs predict post-traumatic growth in the bereaved Better quality of death is associated with higher post-traumatic growth	9/32
*Thomas, Hudson, Trauer, Remedios & Clarke, 2014 *[48] Risk factors for developing prolonged grief during bereavement in family carers of cancer patients in palliative care: A longitudinal study	Longitudinal Prospective Time 1: before the patient's death Time 2: 183 days after the patient's death Time 3: 396 days after the patient's death	Palliative care services, Melbourne, Australia *N* = - 301 at Time 1 - 167 at Time 2 - 143 at Time 3 Woma*N* = 76.7% Spouse = 50.9% Cancer	Self-esteem, family support, financial impact, impact on the organization, impact on health, (Caregiver Reactions Assessment) Partner dependency (Bereavement Dependency Scale) Social support (Multidimensional Scale of Perceived Support) Family functioning (Family Environment Scale) Optimism (Life Orientation Test) Preparedness for Caregiving Scale Perceived competence for the caregiver role (Caregiver Competence Scale) Complicated mourning before loss (PG-12) Anxiety and Depression (Hospital Anxiety and Depression Scale) Complicated mourning (PG-13) Demoralization Scale Post-traumatic stress disorder (PTSD checklist)	In step 1, as a caregiver, living with the patient, being the patient's spouse, not having completed high school, and the patient's young age are so many predictors, at times 2 and 3, symptoms of complicated grief During step 1, as a caregiver, the fact of experiencing anxiety-depressive symptoms, symptoms of complicated grief, and having a low level of optimism, are so many predictors, at step 2 and 3, symptoms of complicated grief. These predictions are improved with the contribution of socio-demographic and psychosocial factors of caregivers In step 1, the caregiver's gender and age, length of care, previous caregiving experiences, and the patient's level of functioning did not predict symptoms of complicated grief at step 2 and 3 Symptoms of complicated grief and a low optima level at time 1 predicted complicated grief at time 3	11/32
*Koop & Strang, 2003 *[49] The bereavement experience following home-based family caregiving for persons with advanced cancer	Transversal Retrospective Mean time between death and protocol completio*N* = 161 days	Capital Health Authority Home Palliative Care Services, Edmonton, Alberta, Canada *N* = 15 Age = 58.5 years Woma*N* = 73.33% Spouse = 60% Cancer	Semi-structured interview guide	Difficult decisions leading to disagreements and harsh words between the caregiver and the patient, the image of the patient's physical decline, and the sense of horror associated with the patient's treatment room contributed to grief adjustment difficulties for the bereaved The fact of having succeeded in accompanying the patient creates a feeling of capability and pride in the bereaved. Likewise, the latter may be made to feel gratitude and relief	7/32
*Yamashita, Arao, Takao, Masutani, Morita, Shima, Kizawa, Tsuneto, Aoyama & Miyashita, 2017 *[50] Unfinished business in families of terminally ill with cancer patients	Transversal Retrospective	Palliative care services, Japan *N* = 642 Age = 61.48 years Woma*N* = 64.6% Spouse = 42.3% Cancer	Unfinished business Depression (Patient Health Questionnaire 9) Complicated grief (Brief Grief Questionnaire)	Families with unfinished business are more likely to develop depression and complicated bereavement (than families with no unfinished business)	10/32
*Chiu, Huang, Yin, Huang, Chien &Chuang, 2009 *[44] Determinants of complicated grief in caregivers who cared for terminal cancer patients	Transversal Retrospective Mean time between death and protocol completio*N* = 271 days	Palliative care services, Taiwan *N* = 668 Age = 42.9 years Woma*N* = 60.6% Spouse = 29.0% Diagnosis of patients: - Lung: 22.8%: - Buccal cancer: 17.1% - HCC: 9.3% - Colon cancer: 8.0% - Esophageal cancer: 7.3% - Pancreatic cancer: 6.2% - Breast cancer: 5.2% - Hypopharyngeal cancer: 4.9% - Gastric cancer: 4.1% - Rectal cancer: 1.8% - Others: 13.2%	Sociodemographic data: - patient: age, sex, cancer, terminal symptoms, duration of treatment, places of care - caregiver: sex, relationship with the patient, religious practices, education, income, medical and psychological history Satisfaction with social support Inventory of Complicated Grief	As a caregiver, being woman, the marital relationship, the parent–child relationship, the absence of religious belief, unavailable family support, a history of mood disorders predispose the bereaved to complicated grief The following factors, namely a length of long-term care, a patient's stay in palliative care and caregivers with a medical history, would be protective factors for complicated bereavement	12/32
*Allen, Haley, Small, Schonwetter, & McMillan, 2013 *[51] Bereavement among hospice caregivers of cancer patients one year following loss: predictors of grief, complicated grief, and symptoms of depression	Longitudinal Prospective Time 1: before the patient's death Time 2: 365 days after the patient's death	Palliative Home Care Services, Florida *N* = 188 Age = 66.41 years Woma*N* = 74% Spouse = 66% Cancer	Caregiver Sociodemographic data: age, sex, ethnicity, education, employment status, relationship with the patient Depression (Center for Epidemiological Studies-Depression) Current feelings of grief (Texas Revised Inventory of Grief) Inventory of Complicated Grief Satisfaction with social support Patient Sociodemographic data: age, sex, ethnicity, education Functional capacities (Palliative Performance Scale) Number of cancer symptoms (Memorial Symptom Assessment Scale)	Time 1 factors, including caregiver's depressive symptoms, fewer patient deficiencies and fewer years of caregiver training predict, at time 2, depression in the bereaved The factors of time 1, namely the depressive symptoms of the caregiver, the young age of the patient, fewer years of training for the caregiver predict at time 2, "worse" bereavement in the bereaved. The deficiencies of the patient and the resources of the caregiver are not significant. Lower social satisfaction correlates with worse grief, but not in the regression analysis Time 1 factors, including the caregiver's depressive symptoms, the patient's young age and fewer years of caregiver training predict at time 2, complicated bereavement in the bereaved. The deficiencies of the patient and the resources of the caregiver are not significant. Lower social satisfaction correlates with complicated grief, but not in the regression analysis There is no effect of gender, ethnicity, employment status, type of relationship, and number of cancer symptoms on depression, bereavement, and complicated bereavement	10/32
*Ferrario, Cardillo, Vicario, Balzarini&Zotti, 2004 *[52] Advanced cancer at home: caregiving and bereavement	Longitudinal Prospective Time 1: before the patient's death Time 2: 91 days after the patient's death Time 3: 183 days after the patient's death Time 4: 365 days after the patient's death	Palliative care team, Northern Italy *N* = - 111 at Time 1 - 96 at Time 2 - 93 at Time 3 - 93 at Time 4 Age = 56.2 years Woma*N* = 66% Spouse = 42% Tumours: - Solid tumours: 97% - Lymphoma: 3%	Performance Status (Eastern Cooperative Oncology Group Performance Status Scale) Family functioning (Family Strain Questionnaire) Personality dimension (Eysenck Personality Questionnaire) Anxiety (STAI XI and X2) Depression (Depression Questionnaire) Subjective satisfaction with life (Satisfaction with Life Scale) Perceptions of physical, emotional and social problems encountered in the last 3 (Caregiver Mourning Questionnaire)	Time factors 1, including being an elderly caregiver (over 61) and having a significant emotional burden, predict a poor adjustment to bereavement in the bereaved Patient's age, social participation, knowledge about the disease, quality of family relationships, and thoughts about death at time 1 have no effect on grief adjustment in the patient. bereaved	11/32
*Ringdal, Jordhøy,* *Ringdal &Kaasa, 2001 *[42] Factors affecting grief reactions in close family members to individuals who have died of cancer	Longitudinal Retrospective Time 1: 30 days after the patient's death Time 2: 91 days after the patient's death Time 3: 181 days after the patient's death Time 4: 396 days after the patient's death	Palliative Medicine Unit, Trondheim, Norway *N* = - 183 at Time 1 - 92 at Time 4 Age = 56.8 years Woma*N* = 68% Spouse = 63% Cancer origin: - Gastrointestinal: 46.4% - Lung: 14.2% - Breast/female genitals:10.9% - Prostate: 8.7% - Others: 19.7%	Sociodemographic data: age, relationship with the patient, child living at home, education, employment status, number of days between diagnosis and death, places of death Current feelings of grief (Texas Revised Inventory of Grief)	Being a woman caregiver, being an older caregiver (> 60 years old) and having lost a young family member are all things that are likely to trigger grieving reactions more important There is no effect of the relationship with the patient, nor of children living at home, nor of employment status, nor of the number of days between diagnosis and death, nor of place of death on adjustment to bereavement	10/32
*Nanni, Biancosino& Grassi, 2014 *[53] Pre-loss symptoms related to risk of complicated grief in caregivers of terminally ill cancer patients	Longitudinal Prospective Time 1: before the patient's death Time 2: 183 days after the patient's death	Hospice, Ferrara, North-East Italy *N* = 60 Age = 60 years old Woma*N* = 75% Spouse = 65% Primary cancer site: - lung: 32.9%; - gastrointestinal: 27.6%, - other: 39.5%	Sociodemographic data: relationship with the patient, caregiver living alone at home Symptoms of complicated pre-loss grief (Inventory of Complicated Grief—Pre Loss) Inventory of Complicated Grief –Structured Clinical Interview	Pre-loss complicated grief criteria including traumatic distress, separation distress, and emotional symptoms appear to be linked to a post-loss complicated grief diagnosis There is no effect of the duration of pre-loss distress on complicated bereavement in the bereaved	9/32
*Götze, Brähler, Gansera, Schnabel, Gottschalk-Fleischer & Köhler, 2016 *[54] Anxiety, depressionand quality of life in family caregivers of palliative cancer patients during home care and after the patient’sdeath	Longitudinal Prospective Time 1: before the patient's death Time 2: 61 days after the patient's death	Ambulatory palliative care teams and palliative care service at the University Medical Center in Leipzig, Germany *N* = - 106 at Time 1 - 72 at Time 2 Age = 65 years old Woma*N* = 69.4% Spouse = 79% Most common diagnosis of sample: cancer of the: - prostate: 17.0%, - lung:14.2%, - pancreas:13.2%, - colon:11.3%	Social support (Oslo Social Support Scale) Quality of Life (Short Form-8) Anxiety and Depression (Hospital Anxiety and Depression Scale)	In time 1, when caregivers experienced high levels of mental distress and insufficient social support, then they were more likely in time 2 to have high levels of anxiety and depression At step 1, when the caregivers were the spouses of patients and had their own weak physical functions, they were particularly depressed at step 2 There is no effect of gender, age, marital status, education, religion, period of care and location of death on anxiety and depression	10/32
*Mori, Yoshida, Shiozaki, Morita, Baba, Aoyama, Kizawa, Tsuneto, Shima& Miyashita, 2018 *[55] ‘‘What I Did for My Loved One Is More Important than Whether We Talked About Death’’: A Nationwide Survey of Bereaved Family Members	Transversal Retrospective Mean time between death and protocol completio*N* = 236 days	Palliative care units, Japan *N* = 678 Age = 61.4 years Woma*N* = 60.9% Spouse = 38.5% Primary cancer site: - Lung: 22.4% - Esophagus, stomach, colon, rectum: 27.3% - Liver, gall bladder, pancreas:18.0% - Breast:4.6% - Head and neck:4.4% - Kidney, prostate, bladder:8.3% - Uterus, ovary:3.8% - Blood, lymph node:1.5% - Other:1.6%	Sociodemographic data of the patient and caregiver Actions in preparation for death Discussion around death Depression (Patient Health Questionnaire-9) Complicated grief (Brief Grief Questionnaire)	Caregivers who had acted and talked about the death before the patient died were significantly less likely to experience depression after the patient died (than those who had neither acted nor spoken) Caregivers who acted before the patient's death were significantly less likely to experience complicated grief after the patient's death, whether or not they spoke (compared to those who neither acted nor spoke)	12/32
*Bradley, Prigerson, Carlson, Cherlin, Johnson-Hurzeler, Kasl, 2004 *[56] Depression Among Surviving Caregivers: Does Length of Hospice Enrollment Matter?	Longitudinal Prospective Time 1: before the patient's death Time 2 = 183 days after patient death	Hospice, Connecticut, United States *N* = 174 Age: < 65 years = 71.8% Woma*N* = 72.4% Spouse = 30.5% Cancer	Sociodemographic data: age, sex, education, annual income, marital status, religious status, relationship with the patient Patient prognosis Number of days in the hospice before death Duration of care provision before enrollment in the hospice Use of services Caregiver Burden (Zarit Burden Interview) Social support Caregiver health Depression (Structural Clinical Interview for DSM)	Caregivers of patients enrolled in a hospice for 3 days or less were significantly more likely to have major depressive disorder at time 2 (than caregivers of those with a longer hospice stay)	12/32
*Gilbar, 1998 *[57] Length of Cancer Patients' Stay at a Hospice: Does it Affect Psychological Adjustment to the Loss of the Spouse ?	Transversal Retrospective Average time between death and completion of the protocol: 336 days for 50.4% of the sample	Hospice, Northern Israel *N* = 134 Age = 64 years old Woma*N* = 61.2% Spouse = 100% Cancer	Sociodemographic data: sex, time in bed, length of stay in hospice, year of diagnosis Psychological distress (Brief Symptom Inventory) Psychosocial Adjustment to Physical Illness Scale Texas Revised Inventory of Grief	A stay in short-term palliative care (1–7 days) had a beneficial effect on the bereavement of the surviving spouse (compared to a long stay, i.e. 8 days or more) Grieving male spouses, a shorter time in bed and a short stay in a hospice would predict less psychological distress	9/32
*Kristjanson, Sloan, Dudgeon &* *Adaskin, 1996 *[58] Family Members' Perceptions of Palliative Cancer Care: Predictors of Family Functioning and Family Members' Health	Longitudinal Retrospective Time 1: before the patient's death Time 2: 91 days after the patient's death	Palliative care services, Manitoba, Canada *N* = - 80 at Time 1 - 64 at Time 2 Age: > 65 years old = 40% Woma*N* = 78.7% Spouse = 55% Cancer	Caregiver Family Inventory of Needs Care Expectations of Family Members (F-Care Expectations Scale) Family Perception of Care (F-Care Perceptions Scale) Family satisfaction with care (FAMCARE Scale) Perception of family functioning (Self-Report Family Inventory) Family Health (Symptoms of Stress Inventory) Patient Symptom Distress Scale (Quality of Life Scale)	The experience of caring for the family during the palliative phase affects the health of family members and the family's ability to function early in the grieving period	7/32
*Liu & Lai, 2006 *[59] Find a way out: bereavement supportin Taiwan hospice	Longitudinal Prospective Time 1: before the patient's death Time 2: 14 days after the patient's death	Mackay Hospice Palliative Care Center at Mackay Memorial Hospital, Taipei, Taiwan *N* = 108 Age = 43 years old Woma*N* = 65% Spouse < 50.8% Cancer	Chinese Anticipatory Grief Scale Mourning (Chinese Perinatal Grief Scale)	Anticipated grief was significantly but moderately correlated with post-death grief Neither age, relationship with the patient, nor gender are factors associated with bereavement	10/32

## Results

### Study characteristics and characteristics of the participants

Of the 18 studies included, 9 were cross-sectional studies and 9 were longitudinal studies. Of the 9 longitudinal studies, 8 included measures before and after the loss of a relative. Sixteen studies were quantitative and 2 qualitative. The studies were international: 11.11% in Oceania (Australia), 38.89% in Asia (Japan, Taiwan, Israel), 22.22% in Europe (Italy, Norway, Germany) and 27.78% in America (Canada, Florida). As per the inclusion criteria, studies referred only to structures and teams that provided palliative care (Palliative Care Units, Identified Palliative Care Beds, Mobile Palliative Care Teams).

The mean score on Downs & Black's "Quality Measurement Checklist" was 10/32, with a median of 10 (rank: 7–12).

A total of 13,539 participants were studied across all selected studies. The number of participants per study ranged from 15 to 9,123. Participants were mainly spouses (55.39%) and women (68.96%). The average age of participants was 58.13 years (calculated from data from 13 out of 18 studies). It is important to note that the results for spouses are mostly for heterosexual couples.

Our systematic analysis of the factors of the palliative phase that influence the grieving experience are grouped under the following categories (identified by our thematic content analysis): 1) caregiving relative; 2) patient; 3) the relational dynamic between thefriends and relatives caring and the patient; and 4) the end-of-life context.

### Impact of factors on the adjustment to grief: relative

#### Socio-demographic factors

Nine studies focused on the effect of gender on the different variables of interest inherent in the experience of the bereaved. In a few studies, gender (being a woman) was associated with poor adjustment to grief [51], symptoms of anxiety and depression [48], and pathological grief [59]. Nevertheless, these results are to be qualified since, according to Hirooka et al. (2017), being a woman could also promote post-traumatic growth [52]. Post traumatic growth is expressed as a positive psychological change undergone as a result of adversity and other challenges in order to achieve a higher level of functioning. In the same way, other authors do not arrive at consensual results as to the impact of gender on the experience of grief. Gender was not associated with a greater risk of pathological grief [54], symptoms of pathological grief [57], adjustment to grief [47, 58], or symptoms of anxiety and depression [49]. Finally, Gilbar (1998), stated that being a man would predict less psychological distress [53].

Five studies focused, among other things, on the impact of age on the experience of grief. Three studies showed that age does not influence grief adjustment [58], symptoms of pathological grief [57], or symptoms of anxiety and depression [49]. However, according to Ferrario et al.(2004), being over 61 years of age was correlated with a poor adjustment to grief [47]. This is a conclusion that Ringdal et al. (2001) also supported: older people would have the most difficulty for adjusting to the loss [51].

The relationship to the ill relative isa variable that has attracted the attention of many researchers. Seven studies presented results on this subject. Being a spouse may predict symptoms of pathological grief [57], pathological grief [59], poor adjustment to grief[47]and significant depression [49]. However, this result isn’t, once again, universal [51, 54, 58]. Finally, losing a parent or losing a child could be a predictor of pathological grief [59].

Three studies analyzed level of education. A low level of education was associated with a risk of poor adjustment to grief [54] and predicted symptoms of pathological grief [57], pathological bereavement [54] and depression [54]. However, in another study, educational level had no relationship with anxiety and depression [49].

Finally, some research focused on scarcely-studied variables: ethnicity, living with the patient, having children at home, marital status, professional status (in employment vs. not in employment) and income. Living with the ill relative would predispose to greater symptoms of pathological grief [57]. There was no evidence in the literature that presence of children in the home [51], ethnicity [54], professional status [54] or marital status [49] is associated with grief, pathological grief or anxiety and depression.

#### Physical factors

An impaired physical quality of life for the caregiver prior to the loss of the close relative would be correlated with a greater anxiety and depressive state [49] and health score [55]. However, caregivers with a medical history would be less likely to develop pathological grief [59].

#### Psychological factors

Different psychological backgrounds have been considered as factors that can influence the experience of grief. Mood disorders and psychotic disorders predicted pathological grief [59]. Separation from parental figures during childhood was positively correlated with feelings of guilt and post-traumatic stress disorder during grief [48]. Past stories of grief predicted psychological distress in times of grief [48]. Finally, anxiety, depression and psychological distress of the bereaved were predicted by a greater number of adverse life events [48].

Significant depressive symptoms during the caregiving period predicted depression post loss [54], poor adjustment to grief [54], symptoms of pathological grief [57]and pathological grief [54]. Anxiety prior to the loss of a loved one increased symptoms of pathological grief [57]. Anxiety and depression experienced by relatives during the palliative phase of the disease predicted anxiety and depression during grief [48, 49]. While losing hope prior to the loss of the loved one is associated with symptoms of pathological grief [57], optimism during this same period promoted adjustment to grief [60].

The guilt felt before the death of a close relative could predict the guilt experienced after the death [48]. The psychological and emotional burden experienced by caregiving relatives during the palliative phase of the loved one’s illness was associated with poor adjustment to grief [47, 60]. In the same way, psychological distress felt before the loss was associated with poor adjustment to grief [48]. A sense of horror associated with the care room (at home) could contribute to difficulties adjusting to grief [46]. Finally, pre-bereavement symptoms predicted poor adjustment to grief [58], pathological grief symptoms [57] and pathological grief [50, 57].

Presence of the family at the time of death [60] and the efforts made by the relatives in connection with the provision of care may promote adjustment to grief [46]. Using avoidance primarily as a coping strategy during the palliative phase of the loved one's illness could lead to the development of post-traumatic stress disorder during the time of grief [48]. Similarly, a high acceptance of responsibility as a main coping strategy prior to the death of the loved one, could lead the bereaved to experience significant psychological distress [48]. On the other hand, difficulties in expressing one's own feelings before the loss of the loved one would appear to be a risk of poor adjustment to grief [60], while the ability to assert oneself and express one's feelings before the loss of the loved one, could promote adjustment to grief [60]. In addition, acting to prepare for the death (defined as something done or performed by families to help achieve a good death of their loved one based on an explicitly or implicitly shared understanding of terminal awareness) of the loved one and talking about it during the pre-bereavement period would reduce depression during bereavement [56]. Acting to prepare for the impending death of the loved one would also reduce the risk of pathological grief [56].

#### Social factors

The body of research on the influence of social support on grief adjustment has similar findings: lower satisfaction with social support during pre-bereavement may be associated with poor adjustment during grief [54, 60], pathological grief [54, 59], anxiety and depression [49]. Conversely, the satisfaction of those around them with formal and informal support during the palliative phase of the disease may promote adjustment to grief [60]. On the other hand, end-of-life discussions (defined as discussions about preferred care or resuscitation measures) between doctors and relatives prior to the death of the loved one may protect against depression during grief and pathological bereavement [61]. Similarly, preparing the family for loss may help adjusting to grief [60]. End-of-life discussions between doctors and relatives prior to the death of the loved one may protect against depression during grief and pathological bereavement [61]. Similarly, preparing the family for loss could help adjusting to grief [60].

#### Existential factors

While the absence of religious beliefs could predict pathological grief [59] and psychological distress [48], their presence may be correlated with post-traumatic growth [52] and adjustment to grief [60]. However, there does not appear to be a relationship between religion and anxiety and depression [49].

### Impact of factors on the adjustment to grief: patient

#### Socio-demographic factors

The age of the ill relative seems to play a role in the grief of those around them. The younger the person in palliative care, the greater the risk of disorders in the adjustment to grief [51, 54], symptoms of pathological grief[57]and pathological bereavement [54].

#### Physical factors

The duration of illness appears to have no effect on life satisfaction after the loss of a loved one, nor on adjustment to grief [47, 51]. More severe disease (general health) may predict anxiety, depression, psychological distress, feeling of guilt [48] and difficulties adjusting to grief [46]. Similarly, when the patient requires less bed rest, the bereaved person’s psychological distress was also less [53]. The presence of symptoms (e.g. confusion, major behavioral changes, cachexia, and uncontrollable pain) may be a risk element for the adjustment to loss [60]. An alteration of the quality of life of patient may also increase the chances of psychological distress, guilt [48] and adjustment to loss for the bereaved [54]. Controlling the pain and suffering of the patient may help relatives to adjust to the grief [60]. However, surprisingly, Allen et al. (2013) showed that the risk of depression during bereavement may be greater if the alteration in the general condition of the loved one was low, then in cases with high alteration [54]. Finally, some studies did not find a significant effect between the health status of the loved one and grief (level of functioning of the loved one and symptoms of pathological grief [57], cancer symptoms and pathological grief, depression and poor adjustment to grief [54]).

#### Psychological factors

Family caregivers’ grief may also be influenced by the attitude of the patient towards their illness. A patient’s denial of the severity of the illness and aggressivity towards their caregivers predicted a poor adjustment to grief, whereas acceptance of the illness and its severity facilitated adjustment to grief [60].

### Impact of factors on the adjustment to grief: relationship between the patient and their caregivers

Communication problems between the loved one and family and friends [60], ambivalent or dependent relationships with the loved one [60], family tensions [60], or difficult decisions leading to disagreements and harsh words between the patient and the relatives [46] could be risky elements for adjusting to grief. Similarly, a significant level of control in the relationship between the loved one and those around them would seem to be correlated with anxiety and depression at the time of bereavement [48]. Less attention to the relationship may predict a sense of guilt during bereavement [48]. Impaired relationships prior to the illness were a risk factor for developing PTSD at the time of grief [48]. Finally, a sense of unfinished business between the patient and their family could predict a poor adjustment to grief and post-loss depression, greater than when business is considered to be "in order" [62]. Conversely, the presence of meaningful relationships between the family and the loved one seems to promote adjustment to grief [60].

### Impact of factors on the adjustment to grief: end of life context

Palliative care plays a role in how family caregivers will adjust to grief. If the ill relative or friend is cared for by a palliative care service this may reduce the risk of pathological bereavement [59]. Palliative care time of less than 3 days is correlated with greater depression during bereavement [63]. Palliative care time of between 1 to 7 days (vs. 8 days and more) was shown to be correlated with better adjustment to grief and less psychological distress during bereavement [53]. However, another study showed that the duration of palliative care had no influence on life satisfaction, nor on bereavement adjustment [47]. In addition, in another study, the patient's departure to a hospital or palliative care service appeared to be a risk element for bereavement adjustment [60]. Nevertheless, the place where the patient died (hospital or home) does not appear to have a significant effect on bereavement adjustment [51], nor on anxiety and depression in the bereaved [49].

Weak practical assistance prior to the loss of the loved one increased the bereaved person’s anxiety, depression and guilt [48]. On the other hand, while a shorter length of care predisposed the bereaved to pathological bereavement [59], it did not appear to have an effect on anxiety and depression in the bereaved [49]. Moreover, previous experiences of care were not a predictor of symptoms of pathological bereavement [57].

Finally, respect for the sanctity of death and the dignity of the loved one promoted adjustment to grief [60]. Quality of death, a global concept taking into account many variables, exerted a positive influence on post-traumatic growth [52]. The quality of death includes the following dimensions: environmental comfort, completion of life, dying in a favorite place, maintaining hope and pleasure, independence, physical and psychological comfort, good relationships with medical personnel, not being a burden on others, good relationships with family, being respected as an individual.

## Discussion

The main objective of this review was to identify characteristics specific to the palliative phase of cancer that influence experiences of the bereaved relative. Results of 18 studies were analyzed to identify predictors that play a role, separately or together, in experiences such as typical grief, pathological grief, depression, anxiety, post-traumatic stress disorder, and post-traumatic growth. As mentioned, in the revised DSM-5, prolonged grief disorder is to be distinguished from major depressive episodes, generalized anxiety disorder and post-traumatic stress disorder. It is for this reason that the discussion is divided into three parts: grief in and of itself, other disorders that are frequently found during the experience of the bereaved, and a part that develops an integrative model incorporating all the factors found in the literature studied in this review.

### Typical grief and pathological grief as a continuum

Certain factors (sociodemographic, physical, psychological, social and existential) inherent in the environment influence the continuum of grief adjustment. It seems that being a woman, being a spouse, a parent or a child, being elderly or having a lower level of education predicts poor adjustment to bereavement. However, not all studies support these results especially with regard to gender, age and relationship [64]. While gender is often listed as a risk factor for pathological bereavement, it must be noted that most of the study participants were women, generating a bias in the results. In addition, due to social norms, men have an increased tendency not to expose their emotions, which again invites us to consider with caution this result concerning a greater vulnerability to loss for women [16]. Regarding age, Sanders [65] introduces a temporal perspective which helps to understand the variability of these results; while young spouses have a higher intensity of mourning immediately after death, it seems, however, easier for them to foresee a better future with new feelings of hope compared to older people [66]. Whether it is about age, gender, or even kinship, we assume that other confounding variables (e.g. attachment, dependence, financial insecurity) can interfere with these and explain this heterogeneity in our results. Finally, educational attainment is associated with type of job and income, this may explain the indirect effect of the level of education on the adjustment to bereavement; a low level of income is a factor which contributes, indirectly, to slowing down the adjustment to bereavement [16]. Financial difficulties can have an impact on social activities by limiting them or even making them non-existent, which could increase the mental ruminations relating to the loss and thus make bereavement more difficult (centered essentially on the loss rather than on recovery) [67].

Depression, anxiety, psychological and emotional strain, psychological distress, feelings of horror associated with the place of care, demoralization and anticipatory grief symptoms are associated with a poor adjustment to grief. It is interesting to come back to the concept of anticipatory grief because it is uncertain. Anticipatory grief is defined as the set of emotional events related to the anticipation of the imminent death of a loved one with the anticipation of emotional pain and life changes related to the upcoming death [10, 10, 30, 68–72]. Sometimes this same concept is used by researchers to describe a pathological process that refers to the detachment and disinvestment of the relationship, as if death had already taken place, while the person is still alive [73]. It is therefore necessary to clearly define this concept in order to limit the confusion around it [30, 68, 70–72, 74]. In this sense, a systematic review of the literature showed that there was a wide variation in the terminology, conceptualization, and characterization of bereavement before death. More than 18 terms and 30 definitions were used to define it; and in many cases, the same term (e.g., anticipatory grief) was defined differently across studies [75]. The authors of this review have attempted to clearly define these concepts so that researchers can rely on uniform constructs to advance this field of research [75]. While many articles not included in our review discuss the impact of anticipatory grief on the experience of grief (perhaps due to definitional differences) [23, 24, 30, 32, 50, 68, 69, 72, 76], our findings range in the same meaning: it is a risk factor. Indeed, anticipatory grief is a stressful experience for caregivers [74]. And we believe that it is a question of intensity: if the manifestations of anticipatory grief are intense, the grief may then become at risk [77]. Thus, when the anticipated grief generates great stress and very intense emotional distress, clinical support for the person experiencing it could be recommended [78].

In this review, it appears that difficulties in expressing feelings predict poor adjustment to grief. Being able to assert oneself and to set up actions in preparation for death of a loved one would be protective. A parallel can be drawn with alexithymia, which contributes to the development of various somatic and psychiatric disorders [79]. In addition, assertiveness is a mature defense mechanism that appears to be a response to emotional conflict and stress, which may explain better adjustment [80]. Finally, preparing for the death of a loved one could be interpreted as a way of coping with the helplessness experienced as it approaches [80, 81].

Preparing oneself for the loss of a loved one positively influences grief. Discussions about the end of life between healthcare professionals and the patient's family and preparation for loss are predictors of better adjustment to bereavement. However, what does this notion of preparation for loss mean and what are its foundations? For example, is this an informative preparation, which can be linked to discussions about the end of life and therefore to the knowledge inherent to the disease? Or is this an emotional preparation? Or pragmatic preparation? Who should it involve: healthcare professionals and caregivers; the caregiver and the loved one; the healthcare professional and patient; or the healthcare professional, patient and caregiver? The 4th edition of the Clinical Practice Guidelines for Quality Palliative Care offers interesting leads [82].

Perceived unsatisfactory social support negatively affects coping with grief. The reverse is also true. Findings on social support can inform data that focuses on religious beliefs. The studies reviewed suggest that the absence of religious beliefs would have a negative impact on adaptation to bereavement and that their presence would promote adaptation to bereavement [48, 49, 52, 59, 60]. Two processes seem to be at work: the fact that religious beliefs provide a system of beliefs and perspectives on which to cope with loss and the fact that religious practice allows inclusion in a social network [16].

As the results indicate, factors inherent to the patient influence the bereavement of those around them. The young age of the patient seems to correlate with poor adjustment to bereavement. This may be explained in that it undermines the bereaved person’s belief in a "just world" since it is difficult to continue to perceive the world as just, orderly, consistent, controllable and predictable when a young person dies [83], or even with regard to the stereotypical feeling that a short life would be unaccomplished.

The patient's attitude towards their illness can be understood both as a risk factor (denial, aggression) and as a protective factor (acceptance) of the adjustment to the bereavement of the loved one. The notion of acceptance can be linked to the acceptance stage described by Kübler-Ross who defined it as the integration of a new reality [84]. This integration phase is often accompanied by defense mechanisms such as dissociation or cleavage which may suggest a form of ambivalence in the patient [81]. To remain ambivalent is to continue to hope (e.g. comfort, freedom, recovery, etc.) [81].

The interaction between a loved one and those around them portends adjustment to grief. An ambivalent or dependent relationship, tensions, disagreements, harsh words, communication problems, and unresolved affairs are predictors of poor grief adjustment. In view of our results, considering adjustment of the bereaved through a systemic prism seems necessary [85–87]. The bereaved cannot be considered in isolation since mourning is synonymous with the loss of an attachment [11, 12, 88]. Thus, like the work of Kissane (1994–2016) and many other authors, advocating systemic therapies that include the patient and their entourage would seem fundamental [89–93].

### Depression, anxiety, post-traumatic stress disorder and post-traumatic growth

Being a woman, being an intimate partner, and having a low level of education seem to be associated with depressive and anxious symptoms for the bereaved. However, these results are again disparate from one study to another. Conversely, being a woman is significantly and positively associated with post-traumatic growth. Childhood separation from parents is associated with guilt and post-traumatic stress disorder. If guilt is part of the grieving process, its intensity can vary depending on the story of the bereaved [94]. It can translate in the bereaved as love for the deceased that did not seem sufficient (e.g., being lacking on certain occasions, hostile thoughts towards the deceased, not having succeeded in saving them, etc.), but it can also reveal a feeling of abandonment in which the bereaved (perhaps unconsciously) blames the deceased for having abandoned them [94]. The feeling of abandonment experienced during mourning can also reactivate past suffering related to breakups, separations and losses [11, 12].

Depressive and anxious symptoms during the caregiving period correlate positively with anxiety and depressive symptoms in the bereaved. To set up actions in preparation for death and talking about the upcoming death appear to be protective factors for depression in the bereaved. Indeed, we know well that the ability of a subject to put into words what they felt has a highly symbolic and subliminal function for the subject who says them and thus facilitates their adjustment [95]. Conversely, strategies, such as avoidance and mutism can lead to the development of post-traumatic stress disorder.

In addition, most studies in this review report that deterioration in the patient's quality of life (especially in the physical dimension) leads to depressive and anxious symptoms for the bereaved. Memories of poor therapeutic control of symptoms hampers the resolution of bereavement [64, 96]. The perceived suffering appears unbearable for the loved one: the suffering must not exist, the helplessness that this generates must not exist, the representation of the end of life as aseptic is undermined [97]. Nevertheless, a study from our review also shows that a lack of change in the general state of the loved one can predict poor adaptation to bereavement. It can be understood as an impossibility for relatives to anticipate the end of life of the loved one due to a lack of body changes (indicator of the severity of the disease) [81]. This could be similar to a "sudden death" (risk factor for pathological mourning) [98].

Too much control in the relationship (need for adhesion, need to perceive actions of the spouse, need to change the spouse, difficulty in accepting a different point of view of the spouse, etc.) exerts a negative influence on the bereaved with anxiety and depression, as does unfinished business that begets depression. Control strategies allow one to protect oneself and prevent the perception of unpleasant sensations and avoids their invasion [99, 100]. This preventive position is very costly, because it maintains a permanent state of psychic and physical tension, which can lead to exhaustion [99, 100]. A conflictual relationship can lead to the development of post-traumatic stress disorder.

Finally, having less help during the period of caregiving can generate anxiety and depressive symptoms in the bereaved. An increased burden has a significant impact on mourning and having help could to decrease the perception of the burden [101].

### Toward integrative modeling of main predictors of the palliative phase of cancer that may influence relatives' grieving experience

**Fig. 2:**
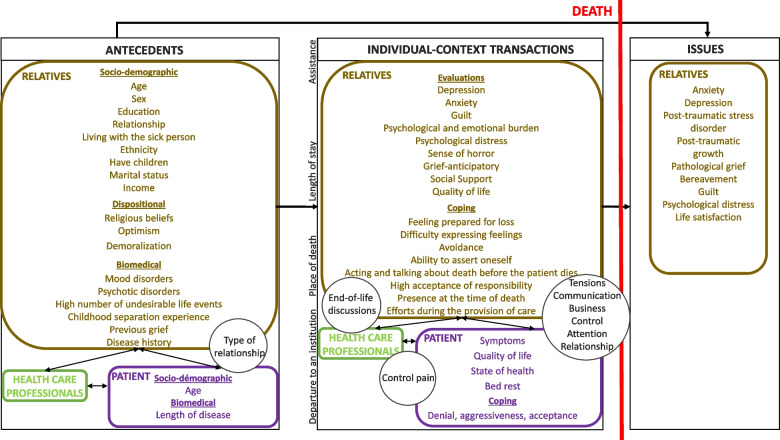
Toward an integrative modeling of main predictors of cancerous palliative phase that may influence relatives' grieving experience

The various factors studied above are represented in a model which aims to be integrative (Fig. 2). This figure is inspired by the Transactional-Integrative-Multifactorial model (transactional because it includes the main processes developed to deal with aversive situations; integrative because it includes components of a different nature (psychological, social, economic, medical, biological, etc.); multifactorial because it includes factors with different functions (predictors, transactions, criteria))[79]while being anchored in a systemic and triadic approach [102].

This model presents the main predictors of adjustment to loss in the family caregiver, identified in this literature review, by organizing them according to two modalities: antecedent variables (environmental, socio-demographic, bio-medical, and dispositional) and transactional variables (evaluative processes, coping strategies, and bio-physiological functioning). It also presents the main markers of adjustment to bereavement explored to date (issues to be predicted). Moreover, it presents the interactions between the three actors involved in the systemic dynamics of bereavement: the loved one at the end of life, the family member and healthcare professional. Between the actors of this system, represented by rectangles, appear other variables modeled by circles (type of relationship, pain control, end-of-life discussions, etc.). These are variables which, in our opinion, can only be linked to one of the constituent actors of the system (patient or relative or health professionals). They are therefore used to model the existing relationships between the three actors in the system (patient-healthcare professional, patient-relative, relative-healthcare professional). For this, the circles are therefore positioned close to the arrow that they serve to qualify; the arrow represents the relationship between the actors in the system. For example, for the antecedents, there is a circle with the qualifier "type of relationship" inserted next to the arrow which makes the link between relative and patient; this means that the type of relationship (ambivalent, dependent relationship) existing between the patient and his entourage may exert an influence on the experience of the bereaved. Sometimes several variables are presented within the same circle. This is a choice on our part to have grouped them with regard to their resemblance (like a thematic synthesis), but this is not an overall factor. For example, for transactional variables, there is a circle with the qualifiers "tensions, communication, businness, control, etc." inserted next to the arrow which makes the link between the relative and patient; this means that the presence of tensions, communication difficulties, unfinished business, or even a significant level of control within the relationship can influence the experience of the bereaved. In order to know how one variable is likely to influence another, it is necessary to refer to the results, since this model aims to be as synthetic as possible.

This model could serve as a reference for clinical practice, aiding in the identification of people who are likely to encounter difficulties in psychological adaptation during their bereavement. Above all, however, it allows us to think about and build interventions for the different actors of this system (relatives, health care professionals, patients). For example, an intervention can be designed for an patient showing aggression, for a caregiver who has difficulty expressing their emotions, or for health care professionals so that a discussion about the end of life can be initiated. While these examples echo individual interventions, this model demonstrates the need to develop systemic interventions. These could, for example, address a disorganized/crisis family system (e.g., tensions, communication difficulties) in light of the severity of the illness. It seems important to be careful not to be too interventionist; it is always useful to allow time for each individual to adapt to the situation they are going through, and this is true for both individual and systemic therapies.

### Methodological limitations

Despite all attempts to make our review as robust as possible, the updating of knowledge and the questions that drive it, many limitations should be highlighted. We will distinguish them according to two types, those relating to the methodology used to conduct our systematic review of the literature and those relating to the included studies. While the methodological approach of our review, which is in line with Cochrane, limits the risks of bias, the exclusion of certain types of studies and our criteria for inclusion and exclusion of the population may result in some bias. Certain contexts were excluded (e.g. other contexts that lead to mourning, other populations, etc.). Finally, even if the methodology used is as empirical as possible, the exhaustiveness of a systematic review is never guaranteed.

In addition, the selected studies include various risks of bias due to their design: recall bias, selection bias, response bias, social desirability bias, absence of psychometric data on certain questionnaires used, small sample size, non-generalizable results, variables not taken into account, correlational effects and not causality, little diversity, high attrition rates, and use of established tools and criteria that are not necessarily those recognized at the present time current [35–45, 48, 51, 52, 57–59]. In addition, from one study to another, we observe significant differences (e.g. variables, pre- and post-loss temporality, etc.) which makes it difficult to compare results from one research to another.

Based on the critical analysis of the integrated studies, these results are based on evidence that is limited. If our results (and our avenues of reflection) are to be taken into consideration, we should also look critically at them. Indeed, our results cannot be considered as a general truth.

Finally, our results cannot be generalized because the couples data is only from a study featuring heterosexual couples only. Moreover, we have not studied the post-death factors that will have a heavy weight in the bereaved's adjustment.

### Clinical implications and implications for research

It is necessary to recognize the unique place that caregivers occupy in the process of supporting a person at the end of life. Though the question of preventive interventions remains with regard to the adjustment to loss [12, 16, 78], in view of our results, there are certain areas of care that should be promoted to support relatives faced with the end of life of a loved one:

- Initiating end-of-life discussions with the caregiver (to allow, among other things, preparation for loss). Bereaved caregivers report wishing they had been better prepared by the care team for the dying process, including the time that it might take [103–106].
- Relieving the overall suffering of the loved one. A study carried out in seven different countries looking at the quality of end-of-life care for cancer patients shows that, in general, the perception of the bereaved concerning the care of the patient was very good [106].
- Relieving the psychic distress of the caregiver (anxiety, depression, guilt, etc.). In the same study, the bereaved report having been sufficiently supported during the last days of the patient's life [106].
- Organizational and material support for the caregiver. A systematic review of end-of-life care evaluation showed that the domains of evaluation related to the environment (related to the room, noise, and comfort of the facility) and caregiver support services are not well explored [107]. If these areas are not well-studied, can we hypothesize that they are perhaps not adequately taken into account by care teams?
- Supporting the communication and the relationship between the loved one and the caregiver in cases of hindrance. Although, to our knowledge, there are no studies evaluating the quality of support for communication in the dyad, a few studies have looked at the various therapies that make it possible to improve dyadic functioning, particularly in terms of communication [91, 93, 108–111]. These interventions have many positive results in reducing distress for patient and caregivers; for patients it's a space that can be used to say goodbye, and relatives’ worries about death are reduced [91, 93, 108–111].

These recommendations are to be thought of according to the singularity of the system we are dealing with: it is not relevant to disorganize a system, thinking "to do well" for the future, by depriving it for the moment of its usual resources [25].While starting work is sometimes important, it is always necessary to assess whether the person or the system are able to tolerate and adapt to changes. Otherwise we run the risk of exceeding the resources of the person and the system and making the crisis worse. In addition, some antecedents, such as mood disturbances, experiences of childhood separation, a large number of adverse life events, age, gender, educational level, type of relationship and low level of optimism can be indicators of “people at risk”. These may be these people who should be supported before, during and after the loss.

It's therefore necessary to continue to explore these variables, adopting methodologies (longitudinal, quantitative and qualitative) that include as little bias as possible, which is a real challenge in this study context. In addition, certain areas of research have been very little explored (e.g. broadening recruitment to account for the plurality of existing caregivers, influence of coping strategies, representations (of death, in particular), fundamental emotions such as fear, guilt and anger, etc.). Future research will have the mission to engage themselves in these unexplored avenues in order to advance knowledge on this subject.

## Conclusion

We have established that there are many factors specific to the palliative phase of cancer that could affect the bereavement of relatives of the patients. These include factors relating to the relatives themselves, to the loved one, to their relationship and end of life context. We have chosen to include these socio-demographic factors and contextual antecedents which are certainly not specific to the palliative cancer phase but which allow us to have a global understanding of the subject. The results of this systematic review of the literature enlighten health professionals on the possible lines of work when supporting someone who is facing the end of life of a loved one. In addition, they deconstruct certain false beliefs. Finally, this review of the literature highlights numerous research perspectives, with, in particular, questions that will bear on the influence of representations of imminent death and death, adjustment strategies and certain emotions-associated fundamentals of life and death.

## Data Availability

All data generated or analyzed during this study are included in this published article.
